# Exploring the Close-Range Detection of UAV-Based Images on Pine Wilt Disease by an Improved Deep Learning Method

**DOI:** 10.34133/plantphenomics.0129

**Published:** 2023-12-15

**Authors:** Xinquan Ye, Jie Pan, Gaosheng Liu, Fan Shao

**Affiliations:** ^1^ College of Forestry, Nanjing Forestry University, Nanjing 210037, China.; ^2^ Co-Innovation Center for Sustainable Forestry in Southern China, Nanjing Forestry University, Nanjing 210037, China.

## Abstract

Pine wilt disease (PWD) is a significantly destructive forest disease. To control the spread of PWD, an urgent need exists for a real-time and efficient method to detect infected trees. However, existing object detection models have often faced challenges in balancing lightweight design and accuracy, particularly in complex mixed forests. To address this, an improvement was made to the YOLOv5s (You Only Look Once version 5s) algorithm, resulting in a real-time and efficient model named PWD-YOLO. First, a lightweight backbone was constructed, composed of multiple connected RepVGG Blocks, significantly enhancing the model’s inference speed. Second, a C2fCA module was designed to incorporate rich gradient information flow and concentrate on key features, thereby preserving more detailed characteristics of PWD-infected trees. In addition, the GSConv network was utilized instead of conventional convolutions to reduce network complexity. Last, the Bidirectional Feature Pyramid Network strategy was used to enhance the propagation and sharing of multiscale features. The results demonstrate that on a self-built dataset, PWD-YOLO surpasses existing object detection models with respective measurements of model size (2.7 MB), computational complexity (3.5 GFLOPs), parameter volume (1.09 MB), and speed (98.0 frames/s). The Precision, Recall, and F1-score on the test set are 92.5%, 95.3%, and 93.9%, respectively, which confirms the effectiveness of the proposed method. It provides reliable technical support for daily monitoring and clearing of infected trees by forestry management departments.

## Graphical abstract

**Figure:**
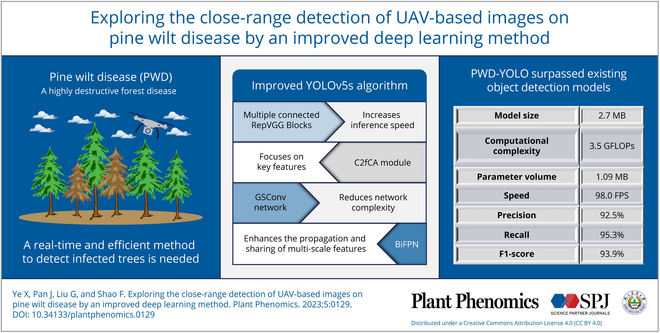

## Introduction

Pine trees (*Pinus* spp.), possessing immense ecological, scenic, and economic significance, face a grave peril in the form of a fatal affliction recognized as pine wilt disease (PWD). Over the past century, PWD has been established as a highly infectious disease caused by the pine wood nematode (PWN; *Bursaphelenchus xylophilus*) [1,2]. While PWD was initially identified in North America [3], it has subsequently been detected in numerous countries across the globe due to its highly contagious nature [4,5]. Among them, East Asia, including countries such as China, Japan, and South Korea, has been recognized as the most severely affected region by PWD [6]. Pine trees exhibit an extensive geographical distribution across China, with a substantial portion of their range overlapping with the favorable habitat for the survival and reproduction of the PWN. Since the initial detection of PWD in 1982 [7], the disease has persistently propagated throughout China for over 4 decades, resulting in the cumulative loss of billions of pine trees and economic losses exceeding hundreds of billions of yuan [8]. According to the announcement by the National Forestry and Grassland Administration (no. 7, 2023), as of February 2023, PWD has caused the mortality of billions of pine trees, affecting 701 counties across 19 provinces on the Chinese mainland, with an affected area exceeding 1.8 million hectares [9]. Consequently, once a healthy pine tree becomes infected with PWD, the prospects for its recovery become exceedingly challenging. Furthermore, without timely detection and prompt implementation of control measures such as cutting down for burning or fumigating, there is a substantial risk of subsequent infection of surrounding pine trees, leading to the widespread dissemination [10]. The evidence suggests that PWD poses a serious and expanding threat in China, characterized by a large scope of impact and high levels of damage. Therefore, there is an urgent need to establish rapid, nondestructive, and highly accurate monitoring and diagnostic methods for precise identification of PWD, laying the foundation for effective prevention and control measures [11].

In PWD-affected regions of China, the *Monochamus alternatus*, as a vector, transmits the PWN by feeding and depositing its eggs on pine trees. Following infection, the pine trees undergo autumn and winter seasons, during which new one-generation *M. alternatus* emerges and flies out from the trees in the subsequent spring, thereby completing a new spread by feeding and laying eggs on the other pine trees again [12]. Research has demonstrated that the most effective approach for epidemic prevention and control involves identifying infected trees and focusing on their targeted removal and immediate on-site disposal through incineration or fumigation during the autumn and winter periods [13,14]. This is because both the pathogenic nematodes and the vector are present in the trees during this time by identifying and chopping down infected trees, followed by implementing measures such as incineration or fumigation to completely eradicate them. Hence, the transmission chain of PWD can be disrupted, leading to highly effective disease prevention and control in theory. Accurate detection of affected trees is crucial for effective PWD management. Traditional ground-based surveys used for identifying diseased trees are characterized by labor-intensive approaches, inefficiency, and relatively high costs, often requiring physically intensive fieldwork deep within forests, making it difficult to accomplish a large volume of work within a limited timeframe [15,16]. Furthermore, within intricate forest environments, certain areas pose challenges for human access, leading to delayed and incomplete information regarding diseased trees. As a solution to this issue, researchers have turned to space remote sensing techniques, including satellite imagery, for PWD monitoring, resulting in significantly enhanced efficiency and reduced costs compared to manual field surveys [17]. This approach is not restricted by geographical limitations and enables the monitoring of extensive pine forest areas, partially mitigating the limitations of manual surveys [18]. Nevertheless, there are limitations associated with space remote sensing. First, it is characterized by long revisit periods, which result in delayed temporal and spatial data acquisition of infected trees, hindering the real-time monitoring requirements for the rapidly spreading PWD. Second, weather conditions have a considerable impact on the data quality, as cloud cover can cause image degradation or even prevent imaging altogether. Third, the low spatial resolution poses a practical challenge, making it difficult to capture detailed features and compromising accuracy [19]. As a complement approach to space remote sensing, aerial remote sensing, using manned aircraft, provides greater flexibility in terms of altitude, angle, and higher spatial resolution for image capture. However, it entails high costs due to the requirement for professional pilots and data acquisition personnel, and the consistency of the acquired data remains a challenge. Although it has improved in terms of resolution compared to space remote sensing, it is still hard to achieve tree-level resolution. Therefore, there is an urgent need for a new remote sensing tool that is cost-effective and highly available, has high spatial resolution, and requires low time and environmental conditions to address the aforementioned issues.

Fortunately, in recent years, close-range remote sensing has emerged as a highly active research field within forest science and remote sensing, which possesses the potential, compared to airborne and satellite-based platforms, to significantly augment the quantity of field observations in both high spatial and temporal domains. This is particularly valuable in addressing the bottleneck associated with calibrating observations from such platforms, which often suffer from a dearth of field references [20]. As an efficient and cost-effective solution in close-range remote sensing, unmanned aerial vehicle (UAV) platforms provide superiorities such as ultrahigh resolution, flexibility, real-time detection, and nonintrusive data collection [21], which make them more advantageous in forest monitoring, particularly in PWD image acquisition, for achieving the individual tree-level resolution compared to previous approaches. The immense potential of UAV remote sensing has been confirmed in forest surveys and parameter extraction [22]. Specifically, several studies have utilized close-range UAV images in combination with traditional machine learning algorithms to investigate and analyze the trees infected by PWD. For instance, Iordache et al. [23] developed a random forest-based classification method using multispectral and hyperspectral images obtained from UAVs to detect PWD-affected trees, achieving an accuracy exceeding 90%. Yu et al. [24] proposed a machine-learning-based approach for monitoring PWD by combining the advantages of UAV-acquired hyperspectral data and LiDAR data, demonstrating higher identification accuracy (73.96%) compared to a single data source (66.86% and 45.56%). Pan et al. [25] explored the impact of spatiotemporal scales on PWD detection accuracy using Jeffries–Matusita and Random Forest algorithms with hyperspectral data from different altitudes. Despite the advancements achieved in monitoring diseased trees through traditional machine learning methods, these studies still rely on professionals to extract image features of the diseased trees, a process that is tedious, subjective, and inefficient. Moreover, this task presents challenges as it is susceptible to the influence of irrelevant background noise in the images, which often leads to the erroneous extraction of nonessential and noncritical feature information, directly undermining the model’s generalization ability and the accuracy of disease tree detection [26].

With the rapid development of artificial intelligence technology in recent years, especially the advancements in deep learning methods, several new algorithms have been proposed. Representative types include 2-stage algorithms such as Fast R-CNN [27], Faster R-CNN [28], and Mask R-CNN [29], which have high accuracy but relatively slower speed, and one-stage algorithms such as the YOLO (You Only Look Once) series [30–32], which have fast inference speed but relatively lower accuracy. These algorithms possess complex neural structures and multiple layers of processing units. Following model training, they can transform simple low-level features such as color and texture into computationally interpretable complex high-level features, such as semantic information, enabling automatic learning, feature extraction, and inference recognition of target objects [33]. In comparison to traditional machine learning methods, these deep learning methods offer advantages such as lower learning costs, rapid detection, and exceptional efficiency, rendering them efficient detection methods [34].

On the other hand, the close-range UAV imagery currently utilized for monitoring PWD primarily includes red–green–blue (RGB), multispectral, and hyperspectral images. Although multispectral imaging offers advantages such as lower cost, simple data processing, and easier implementation, it suffers from limited spectral information for further in-depth research in PWD detection. In addition, its performance instability often leads to poor accuracy, which is also a challenge that needs to be addressed. By contrast, hyperspectral imaging, characterized by numerous narrow bands, offers higher spectral resolution and richer spectral features compared to multispectral imaging. It provides more detailed information by leveraging the distinct spectral responses exhibited by pine trees at different stages of infection, associates this information with each stage to detect early-stage infected trees that may not show obvious signs of infection, and enables the differentiation of different infection stages for PWD [35,36]. However, the acquired hyperspectral data are characterized by high redundancy, often accompanied by dozens or even hundreds of spectral bands. It requires skilled professionals to meticulously preprocess and reduce the dimensionality of the data using powerful computational resources and specialized software. The purpose is to reduce data storage requirements and shorten processing time. Any erroneous operation can lead to unreliable results. It is also crucial to acknowledge that hyperspectral imaging equipment is more susceptible to environmental factors, including light conditions and atmospheric interference. Variations in light intensity and color temperature directly affect the color and contrast of the images, and atmospheric particles obstruct the propagation of light, resulting in signal attenuation. As a result, frequent calibration based on the prevailing environmental conditions to obtain accurate spectral information is necessary, and this practice considerably affects data acquisition efficiency. Furthermore, hyperspectral imaging equipment is mostly bulky and expensive and incurs substantial maintenance costs.

Considering the affordability of implementation and the real-time requirements for PWD monitoring, RGB true-color images captured by UAV platforms have garnered early attention in PWD detection research [37,38]. In this approach, complex convolution and pooling operations are used to extract features of PWD-infected trees from the images, including local features such as texture, color, and morphology, as well as global features. These features are then input into a neural network model for training and modeling, enabling it to learn the general characteristics of PWD-infected trees and detect them in the images. Compared to approaches relying on spectral features, this offers several advantages, including low cost, simple data processing, and flexibility in usage. Deploying it on UAVs has shown its applicability for PWD detection under various environmental conditions, greatly enhancing the practicality and accessibility of this method. Given the highly infectious and detrimental nature of PWD, prompt removal of infected trees is essential to break the transmission pathway. Therefore, there is an urgent need for a cost-effective, convenient, and feasible method that enables forestry management agencies to swiftly identify infected trees in forest areas. Such a method would provide reliable technical support for subsequent cleaning operations and ultimately aid in mitigating the epidemic. Previous studies have demonstrated that the detection of PWD-infected trees at the tree level can be achieved by UAVs equipped with RGB cameras in combination with deep learning algorithms, which have been demonstrated to be best suited for pure pine forests and areas with low disease prevalence. However, PWD-infected trees often occur in nonisolated forest environments, where strong interference from complex backgrounds, including bare ground, dead trees, and red broadleaf species, is prevalent. Under such conditions, the method faces challenges in extracting features from PWD-infected trees, resulting in suboptimal detection performance [39]. In response, attempts have been made by researchers to improve algorithms and better mitigate interference caused by backgrounds, aiming to enhance the detection accuracy of PWD-infected trees. Deng et al. [40] improved Faster-RCNN by optimizing loss functions, anchoring settings in the Region Proposal Network (RPN), and increasing model size, ultimately improving PWD detection accuracy from 66.2% to 89.1%. Hu et al. [41] combined the advantages of deep convolutional neural networks, deep convolutional generative adversarial networks, and AdaBoost algorithm to remove complex backgrounds such as soil, roads, and rocks, proposing a PWD recognition method that effectively reduces false positives and false negatives, thus improving accuracy. Qin et al. [42] designed a spatial information preservation module to mitigate the loss of detailed information during the convolutional process, proposing the spatial context attention network, which has been proven to suppress background interference and extract PWD-specific features, achieving high-precision identification. Nevertheless, most of these studies have achieved desirable results by increasing model size and complexity and paying less attention to model lightweight. The large model size and slow inference speed make it challenging to adapt to resource-constrained embedded devices, such as consumer-grade UAVs, limiting their practical applicability.

In summary, despite the notable progress in previous studies related to the detection of discolored trees infected with PWD, several challenges remain. To begin with, the existing models are primarily effective in pure forest settings without interference, but their detection performance significantly deteriorates in complex environments, such as mixed forests comprising both coniferous and broad-leaved trees, and under various interferences. In the next place, the well-trained models are generally too large and complex to be deployed on consumer-grade UAVs or other devices that lack sufficient computing resources. In the third place, some previous related studies have attempted to optimize the model’s performance through algorithmic improvements, but the detection accuracy and speed cannot be balanced simultaneously. In light of these issues, our research objectives are as follows: (a) validate the efficacy of using only close-range UAV-captured RGB images for detecting PWD in complex forest environments; (b) explore an improved deep learning approach for achieving the detection of PWD-infected trees in various forest environments containing red deciduous species, dead trees, bare ground, and more. In comparison to existing methods, it exhibits a lighter model, faster inference speed, and higher detection accuracy.

The main contributions of this paper are as follows:

1. A high-resolution dataset of PWD-infected trees was established, which includes various forest complex conditions such as dead trees, red broadleaf species, bare ground, and other background noise interference.

2. PWD-YOLO, a PWD-infected tree detection algorithm with speed and accuracy, was proposed. The design is as follows: (a) A lightweight backbone network was designed, which is formed by the concatenation of simpler RepVGG. This design avoids the additional computational burden caused by the flow between different networks and further compensates for the drawbacks of a multibranch architecture through its unique residual design. As a result, the objective of reducing network parameters and improving inference speed is achieved. (b) To address the drawback of channel information loss caused by low computation, the feature fusion network of the model proposed the C2fCA module that possesses richer gradient flow information and introduced the GSConv network, replacing the original algorithm’s C3 module and regular convolutions, striking a balance between speed and accuracy. (c) The Bidirectional Feature Pyramid Network (BiFPN) strategy was used to enhance the propagation and sharing of information among PWD-infected trees at multiple scales in the network.

3. By comparing it with other mainstream object detection algorithms and evaluating it on the test set, the performance of PWD-YOLO was demonstrated, along with its practical application potential.

## Materials and Methods

### Study area and data acquisition

The research area is located in Laoshan National Forest Park, Pukou District, Nanjing City, Jiangsu Province (32°02′48″ to 32°09′48″N, 118°24′34″ to 118°40′59″E). With a total area of approximately 80 km^2^ and a forest coverage rate of 80%, it is the largest national forest park in Jiangsu Province. It has existing forest land of approximately 6,266 hectares, primarily comprising various pine tree species, including masson pine (*Pinus massoniana*), torch pine (*Pinus taeda*), slash pine (*Pinus elliottii*), and black pine (*Pinus thunbergia*). The area belongs to the subtropical monsoon climate zone, with an annual precipitation of 1,000 mm and an average temperature of 15.3 °C throughout the year. The 4 seasons are distinct, and the climate is pleasant, making it particularly suitable for the breeding of the vector, the *M. alternatus*, responsible for the spread of PWD. As such, it is one of the key monitoring areas for PWD in Jiangsu Province. Given the complex forest structure and environmental conditions in this area, which are highly conducive to the present study, the data were collected here for model training and validation. In addition, a portion of the data was obtained from Qingyuan District, Ji’an City, Jiangxi Province (26°38′ to 27°10′N, 114°56′ to 115°30′E). In recent years, this area has experienced severe PWD outbreaks and exhibits notable differences in geographical location, topography, and forest structure compared to Laoshan National Forest Park. Therefore, it was used to evaluate the generalization ability of the improved model.

The field investigation was conducted from 5 to 17 October 2022. A total of 300 pine trees (suspectedly infected trees) were selected for measuring their growth conditions (Table 1), and the needle samples were brought back to the laboratory and confirmed to be infected with PWD through methods such as the Baermann funnel technique and molecular identification. According to previous studies [43], 4 stages of infected pine trees were defined. The middle and late stages that had an obvious visual contrast were the focus of this study as it was during this period that both the vector and PWN were present within the infected trees. By removing these infected trees from the forest, the transmission pathway can be blocked. Therefore, these trees are the primary targets for felling and further incineration or fumigation.

**Table 1.** The growth situation of pine trees in the training area and verification area

Measurement items	Maximum	Minimum	Mean	Standard deviation	Range
H (m)	19.5	3.6	11.2	3.3	15.9
CD (m)	8.5	2.7	3.7	2.1	5.8
CBH (m)	4.8	1.3	3.3	1.2	3.5
DBH (cm)	32.7	10.5	19.4	6.1	22.2

H, tree height; CD, crown diameter; CBH, crown base height; DBH, diameter at breast height.

**Fig. 1.**
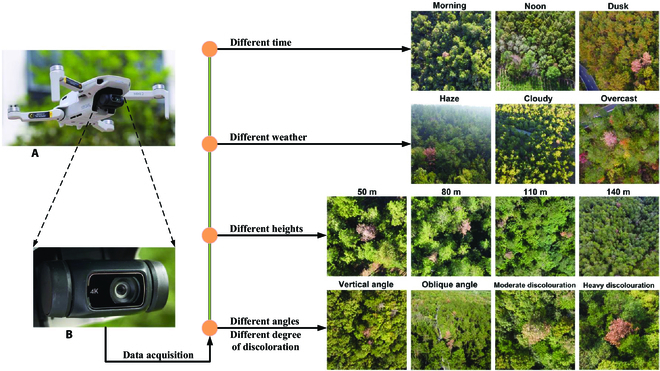
Data acquisition under different conditions. (A) DJI Mini 2 UAV. (B) Complementary metal oxide semiconductor camera with RGB lens.

Image data used for model training and validation were collected in Laoshan National Forest Park, Nanjing City, Jiangsu Province, during the middle stage and late stage of the PWD outbreak (October to December 2022), using a DJI Mini 2 quadcopter produced by DJI Innovation Technology Co. Ltd., a Shenzhen-based company. The UAV weighs 249 g and measures 245 × 289 × 56 mm. It can climb at a maximum speed of 5 m/s, fly horizontally at a maximum speed of 16 m/s, and reach a maximum altitude of 4,000 m. Equipped with a complementary metal oxide semiconductor RGB camera with an effective pixel count of 12 million, the UAV can endure up to 31 min of flight time and has a wind resistance rating of level 5. These features make it a portable and versatile tool that ensures the smooth completion of data collection tasks. Image acquisition was conducted at different times of day (morning, noon, and dusk), under varying weather conditions (haze, cloudy, and overcast), at different flight heights (50 to 140 m), from various shooting angles (vertical and oblique), varying degrees of discoloration (moderate and heavy), and in areas with different forest densities and structures (Fig. 1) to ensure the diversity of the dataset and enable the trained model to possess excellent generalization ability and robustness, avoiding overfitting caused by limited data and enabling efficient detection of PWD in diverse forest environments. Because of the sporadic distribution of infected trees in the forest, using flight route planning software to obtain complete orthophoto imagery for the entire area would be inefficient and costly. We solely required data imagery under the specific conditions and environments mentioned earlier. Therefore, the manual control of flight routes and shutter strategies was chosen to ensure the acquisition of high-quality data and efficient work. Over 30 flight missions were conducted during this period, resulting in the acquisition of more than 7,000 high-resolution (1,902 × 1,080) JPG images containing middle and late stages of infected pine trees, preserving more texture and morphological features of PWD-affected trees for subsequent model training. Furthermore, it is worth noting that to further evaluate the generalization ability of the proposed model in real-world scenarios and assess its robustness in unfamiliar environments, 408 image data were collected in Qingyuan District, Ji’an City, Jiangxi Province, from 20 to 22 October 2023.

### Dataset

**Fig. 2.**
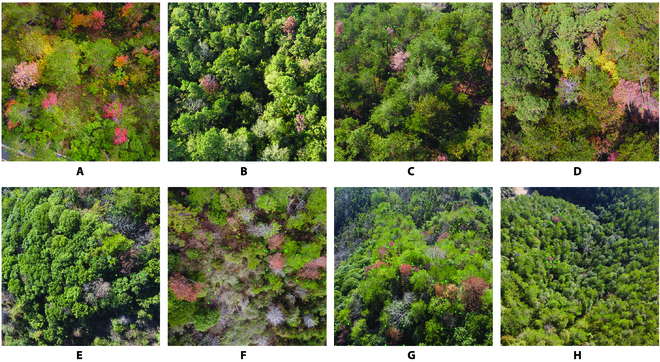
Data examples in different environments annotated by LabelImg. (A) Red broad-leaved trees and bare land. (B) Red broad-leaved trees. (C) Target occlusion. (D) Red broad-leaved trees and target occlusion. (E) Dead trees and incomplete targets. (F) Dead trees and target occlusion. (G) Multiscale and dead trees. (H) Small target.

To improve the quality of the dataset, effective data that are clear and undistorted were selected, while ineffective data that are blurry and severely distorted were eliminated from the raw data obtained. Eventually, the dataset of 6,308 images of discolored trees infected with PWD, captured under various environmental conditions, was divided into a training set of 5,147 images (approximately 82% of the total) and a validation set of 1,161 images (approximately 18% of the total), based on the location of these images acquisition and the requirements of subsequent experiments. It is important to note that the training set and validation set were obtained from different locations within the study area, and they do not intersect. In addition, an independent test set consisting of 408 images was created, which was not used for training the model but solely for testing its generalization capability. The dataset was annotated using the LabelImg image annotation software, resulting in a total of 39,094 samples. Among them, 34,177 were training samples, and 4,917 were validation samples, with the sample labels saved in YOLO format TXT files. The dataset contains samples captured under various complex conditions, such as small targets, dead trees, multiple scales, occlusion, bare ground, and red broad-leaved tree species with strong similarity. These samples often pose challenges to the model in terms of erroneous detection and omission due to the similarity in color between PWD-infected trees and the background environment or the indistinct features during the identification process, as shown in Fig. 2.

### Methods

#### Model overview

Object detection is a popular task in recent years, aiming to identify and locate specific objects or entities within an image. YOLOv5, an end-to-end object detection algorithm, was developed by the team at Ultralytics LLC [44]. On the basis of the different depths and feature map widths of the trained model network, it is divided into 4 versions: YOLOv5s, YOLOv5m, YOLOv5l, and YOLOv5x. All of these versions detect and locate objects through direct regression from the original image, and as the network depth and feature map width gradually increase from the “s” version to the “x” version, the number of parameters constantly increases, detection accuracy continuously improves, and detection speed progressively slows down. Because of its smaller size, YOLOv5s is considered to be more suitable for practical scenarios than the other 3 models. However, it should be noted that when directly applied to detect PWD-infected trees in complex forest environments, lower detection performance and slower speed are exhibited by YOLOv5s. This can be attributed to the following reasons: In terms of data, the presence of background noise such as red broadleaf species, dead trees, and bare ground in the forest can lead to false recognition. In terms of algorithm architecture, the original YOLOv5 was not specifically designed for the characteristics of PWD-infected trees, resulting in a tendency to miss detections in scenes where the feature representation is not prominent. In addition, for small-scale embedded devices such as consumer-grade UAVs, the original model size remains unfriendly and requires further improvement to meet the practical needs of forestry management.

**Fig. 3.**
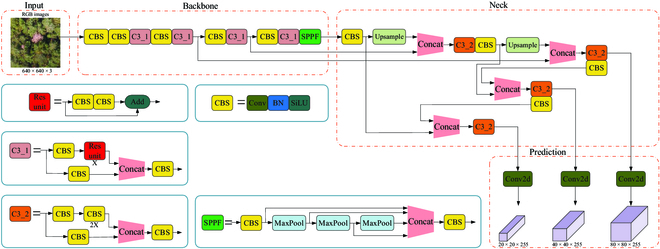
Network architecture of YOLOv5.

Figure 3 shows the YOLOv5 network structure diagram, which is composed of 4 parts: input, backbone, neck, and prediction. Within this range, a crucial role is played by the input layer in processing the image and generating anchors. It scales the images to a unified size for input into the network and adaptively generates anchor boxes of different sizes. On the basis of this foundation, during the model training process, it outputs predicted boxes that cater to potential targets’ sizes, thereby fulfilling the detection of various scale objects, particularly small targets. Features at various scales are extracted by the backbone network using multilayer convolution. The neck fuses feature information from different scales, which is conducive to improving feature representation and enhancing spatial information, thereby enhancing the accuracy and robustness of the model and reducing the risk of overfitting. The generation of valuable information including the categories, probabilities, and positions of the detected targets is facilitated by the prediction layer through the utilization of 3 prediction branches. Each prediction branch is tailored to different scales of targets, as they possess 3 anchors of varying sizes. This contributes to enhancing the ability to predict PWD-infected trees at multiple scales and reducing missed detections.

#### Backbone

The performance of the model after training can be directly determined by the quality of the network structure of deep learning algorithms. However, any superior network structure needs to be combined with specific application scenarios. Because of the typically bulky size of existing object detection models, deploying them on small-scale embedded devices with limited computing power, such as consumer-grade drones, poses a crucial challenge in terms of inference speed, making it difficult to meet the requirements of real-time detection. Nonetheless, performing real-time detection on PWD-infected trees within forest areas necessitates models that satisfy the prerequisites of being compact, fast in inference speed, and having fewer parameters. The overall performance and size of the model are significantly influenced by the backbone network.

**Fig. 4.**
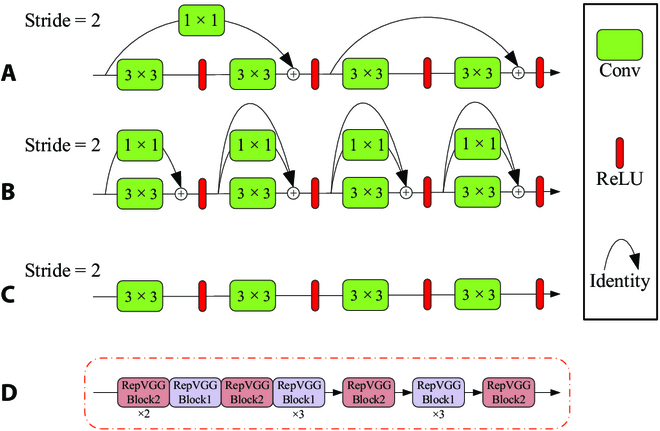
Module schematic of RepVGG. (A) ResNet. (B) RepVGG training. (C) RepVGG inference. (D) Backbone network composed of multiple connected RepVGG Blocks.

An improved version of VGG, RepVGG [45], has a structure similar to VGG, consisting of 3 × 3 convolutions and rectified linear unit (ReLU). It is simple, has few parameters, and is suitable for deployment on mobile devices with limited computing resources. Structural reparameterization technology, inspired by ResNet, is applied to this network, enabling the training model to have a multibranch topology structure. During training, multiple identical or different modules can be created by splitting a module, and equivalent modules can be formed by fusing multiple previously split modules during inference. In ResNet, the information flow is modeled as *y = x + f*(*x*), and *f* is learned in a residual manner. If the channel numbers of *x* and *f*(*x*) do not match, *y = g*(*x*) + *f*(*x*), where *g*(*x*) adjusts the channel numbers of the residual part to match *f*(*x*) via a 1 × 1 convolution. The effectiveness of this approach in improving model performance has been verified by ResNet. In RepVGG, this idea is also applied by modeling the information flow as *y = x + g*(*x*) *+ f*(*x*). During training, 2 types of residual structures are used in the network architecture: The first type is a 1 × 1 convolution residual branch, and the second type contains not only a 1 × 1 convolution residual branch but also an identity residual branch. Simple residual structures were initially used in the model, but as the network deepens, the residual structures become more complex, aiming to obtain robust features while addressing the gradient vanishing problem in deeper layers. During inference, the trained modules are converted into a single 3 × 3 convolution and then used through reparameterization. Therefore, a lightweight backbone network (Fig. 4) was designed, composed of multiple connected RepVGG Blocks, instead of the relatively complex structures of the original YOLOv5, containing C3 and SPPF (spatial pyramid pooling-fast). This design strategy avoids the additional computational burden caused by the flow between different networks and addresses the drawbacks of a multibranch architecture and enables the backbone network to efficiently extract key features of PWD-infected trees while minimizing computational costs and maximizing inference speed.

#### C2fCA module

Object detection is treated as a regression problem by YOLO, and targets are directly detected and located from images through regression. The regression approach used inevitably makes it difficult to effectively distinguish between foreground and background areas in the input image, leading to missed detections and false detections and ultimately resulting in a decrease in accuracy. The detection of the discolored pine trees infected by PWD in UAV images taken under natural conditions is the research subject of this study. A large amount of interference, such as exposed ground, red broad-leaved tree species, and dead trees, is contained in these images obtained via UAVs, making the background complex and diverse. A challenge is posed for the object detection task by these interferences, as indiscriminate attention is paid by the model network to all feature maps extracted, making it difficult to concentrate on the key feature information during training.

**Fig. 5.**
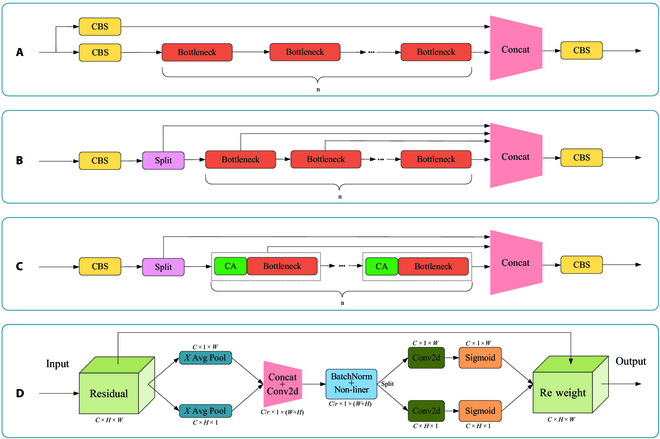
Network structure of C3, C2f, and C2fCA modules. (A) C3 module. (B) C2f module. (C) C2fCA module. (D) Network structure of CA mechanism. *C* × *H* × *W* represents the 3-dimensional size of the image; *C* × 1 × *W* and *C* × *H* × 1 refer to the sizes of the clustering feature maps; *X* Avg Pool represents one-dimensional horizontal global pooling, and *Y* Avg Pool represents one-dimensional vertical global pooling; Concat denotes channel fusion; Conv2d denotes 2-dimensional convolution; *C*/*r* × 1 × (*W* + *H*) represents the intermediate feature map that encodes spatial information in both horizontal and vertical directions simultaneously; BatchNorm denotes the Batch Normalization algorithm; Nonliner refers to nonlinear regression; Sigmoid denotes the Sigmoid function.

In addition, in the original YOLOv5 structure, the C3 module, which consists of 3 standard 3 × 3 convolutions and plays a role in learning target features in the network, was not specifically designed for the features of the discolored pine trees infected by PWD, resulting in redundancy in some network modules during the model learning process. This causes the model to become cumbersome and prevents it from fully utilizing its performance, leading to unnecessary computational costs. The C3 module is shown in Fig. 5.

The C2f [46], a module with rich gradient flow information that adjusts the corresponding channel numbers for different scale models to enable the capturing of more texture feature information, has many skip connections and split operations added to its network to make the model more lightweight and compensate for the shortcomings of the original C3 module in terms of model lightness. The C2f module is shown in Fig. 5.

The ability to capture features from 2 channels, considering both channel information and location information simultaneously, is possessed by coordinate attention (CA) [47]. It forms a set of direction-sensitive and position-sensitive feature maps that can be used to complementarily enhance effective feature information, helping the model network to quickly focus on the target’s texture features. In addition, the residual design is applied in the CA mechanism network, avoiding the problem of gradient vanishing and making the network more efficient. The structure of the CA mechanism is shown in Fig. 5.

To improve the model network’s ability to focus on effective feature information and enhance the target feature information (such as texture, color, etc.), the C2f module is combined with the CA mechanism to design a C2fCA module with richer gradient flow information and stronger attention to key areas. This module was used to replace the C3 module in the original YOLOv5 feature fusion network. It ensures that more correct and important feature information is retained, while redundant features are ignored. This effectively enhances the fitting and expression abilities between modules while also maintaining the lightweight nature of the network. The network of the C2fCA module is shown in Fig. 5.

#### GSconv

**Fig. 6.**
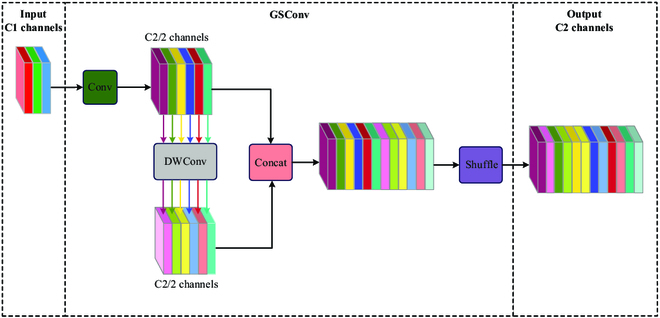
Structures of GSConv.

Shaping a more lightweight network architecture, one of the mainstream methods is achieved through introducing depthwise separable convolution (DSC). Although DSC has efficient computational capability with lower computational and parameter complexity, channel information is lost during calculation, resulting in inferior feature extraction and feature fusion capability compared to standard convolution (SC). The shortcomings of DSC are addressed by GSConv, which shuffles channel information via a combination of SC, DSC, and shuffle. Shuffle, a channel mixing method proposed and validated in ShuffleNets [48], is effective in channel information interaction, enabling the information generated by SC to be completely mixed into the output of DSC, thereby achieving the purpose of channel information interaction. An approach to channel mixing is applied by GSConv. Specifically, the feature map with input channel number C1 performs DSC on half of the channels and SC on the remaining half. Then, the 2 are connected and feature concatenated. Then, the information generated by SC infiltrates each part of the information generated by DSC through the shuffle, and the output feature map has channel number C2, balancing the model’s performance and computational cost. The goal of lightweight models is achieved by introducing GSConv into the YOLOv5 model network. The structure of GSConv is shown in Fig. 6.

#### BiFPN

**Fig. 7.**
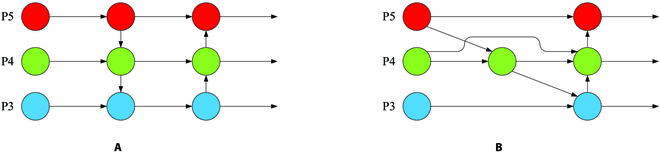
Structures of PANet and BiFPN. (A) PANet. (B) BiFPN.

The PANet network, an improvement on the Feature Pyramid Network (FPN) [49], is used in the original YOLOv5 to add bottom-up information enhancement to FPN and retain more shallow features of the targets. However, retaining an excessive amount of shallow semantic information can lead to the loss of deep semantic information, resulting in a network that is both complex and inefficient. BiFPN was initially proposed in EfficientDet [50], and its structure adds cross-scale connections through the introduction of weights to gather feature information from different scales, retaining both shallow and deep semantic information in the network. This ultimately improves model accuracy and makes the network simple and efficient. Inspired by this idea, the same-scale skip connections were added to the feature fusion network of PWD-YOLO, and nodes with low feature contributions were removed, greatly improving the efficiency of feature fusion in the network. The BiFPN structure is shown in Fig. 7.

#### Improved YOLOv5s model

**Fig. 8.**
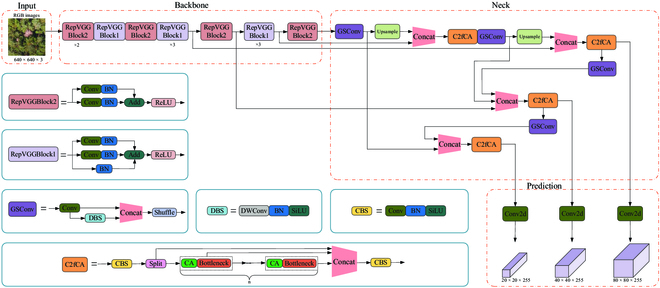
Network Architecture of PWD-YOLO.

On the basis of the YOLOv5s model, it was optimized and improved according to the application scenario and practical needs in forestry management. Specifically, a lighter backbone network was designed, composed of multiple connected RepVGG Blocks, which avoids the additional computational burden caused by traffic flow between different networks and further compensates for the shortcomings of the multibranch architecture through its unique residual design. As a result, the network parameters are reduced, and the inference speed is improved. Furthermore, to address the drawback of partial feature information loss due to the simplified backbone, the C2fCA module was designed in the feature fusion network to enrich the gradient flow information and focus more on key regions, and the GSConv network was introduced to replace the original C3 module and conventional convolutions, which helps the model extract feature information of PWD-infected trees in complex backgrounds and strong interference, striking a balance between speed and accuracy. In addition, to address the issue of varying PWD-infected tree scales caused by distinct differences in forest terrain, leading to false positives and false negatives, a weighted BiFPN strategy was introduced to enhance the propagation and sharing of multiscale features. With the aforementioned designs, a lightweight and efficient model named PWD-YOLO was obtained, which has a smaller model size, fewer parameters, and a simpler network structure while maintaining high detection accuracy in contrast with the original model. The PWD-YOLO network structure is shown in Fig. 8 and consists of Input, an improved lightweight backbone network, a feature fusion module with richer gradient flow information, and a Prediction module.

### Implementation and assessment methods

#### Experimental platform and parameter settings

In this study, the image size of the model network input was set to 640 × 640 pixels, and it was trained for 150 epochs with a batch size of 32 and Num workers of 16. The hyperparameters in the experiments were set as follows: The initial learning rate was set to 0.002, and the cosine annealing algorithm was used for learning rate decay. The optimizer used was Adam, with a momentum of 0.937. The threshold for nonmaximum suppression was set to 0.5, the intersection over union was set to 0.5, and the confidence threshold was set to 0.55. To prevent overfitting of the model, data augmentation was performed using the 4-mosaic method before image input. This involved randomly scaling, rotating, and cropping 4 images, which were then combined into one image to increase the number of training samples. All models involved in this study were run on the same device, and the specific configuration and experimental environment are shown in Table 2.

**Table 2.** Hardware configuration and experimental environment.

Hardware	Configuration	Environment
Operating system	Ubuntu 18.04	Python 3.8
CPU	Intel Xeon 8225C @2.5GHz	PyTorch 1.9.0
GPU	NVIDIA GeForce RTX 2080Ti	CUDA 12.0
RAM	43 GB	**—**

#### Evaluation indicators

As this research is aimed at detecting discolored trees infected with PWD in existing forest areas, the ultimate goal is to solve practical problems in daily work. Therefore, real-time and accurate detection is required, and the parameter size and model size should also meet application requirements. The main evaluation indicators of the model detection performance, including Precision (P), Recall (R), mean average precision (mAP), and F1-score, are determined. In addition, the model size and network complexity are measured by parameters and floating point operations (FLOPs), and the speed of the model is measured by frames per second, which represents the number of images processed per second. The calculation methods these indicators are shown in Eqs. 1 to 7:

FLOPs=2HWKhKwCinCout+Cout (1)

$$\text{Parameters}=K_{h}K_{w}C_{\text{in}}C_{\text{out}}$$ (2)

$$\mathrm{FPS}=\frac{1}{t}$$ (3)

$$P=\frac{\mathrm{TP}}{\mathrm{TP}+\mathrm{FP}}$$ (4)

$$R=\frac{\mathrm{TP}}{\mathrm{TP}+\mathrm{FN}}$$ (5)

$$\mathrm{mAP}=\frac{\sum_{i=1}^{N}\;AP_{i}}{N}$$ (6)

$$\text{F1}=\frac{2\mathrm{PR}}{P+R}$$ (7)

In Eqs. 1 and 2, *H*, *W*, and *C_in_* denote the height, width, and channel of the input feature map, respectively, while *K_h_* and *K_w_* denote the height and width of the convolution kernel, respectively, and *C_out_* denotes the number of output channels. In Eq. 3, *t* represents the time required to process a single image. In Eqs. 4 and 5, *TP* denotes true positive, *FP* denotes false positive, and *FN* denotes false negative. In Eq. 6, *N* represents the number of categories, and mAP@0.5 is selected as the accuracy evaluation metric in this study, which refers to the precision of detecting images when the intersection over union is 0.5.

## Results

### Contrast experiments

To demonstrate the effectiveness of the proposed PWD-YOLO, it was compared with mainstream object detection models, and the experimental results are shown in Table 3. In terms of model lightweightness, the model size, computational complexity, and parameter size of PWD-YOLO proposed in this paper are 2.7 MB, 3.5 GFLOPs, and 1.09 MB, respectively, which are the smallest among all models, indicating that the model network is the simplest. Second, PWD-YOLO is the fastest among all models, with the frames per second of 98.0, which is 42.4, 26.6, 28.1, 22.8, 30.0, 35.9, and 7.1 higher than Faster R-CNN, SSD, YOLOv3, YOLOv4, YOLOX, YOLOv7, and YOLOv5, respectively. Regarding detection accuracy, PWD-YOLO’s mAP@0.5 is the highest among all detection models, reaching 87.7%, and its F1-score is only slightly lower than Faster R-CNN at 85%, reaching 83%.

**Table 3.** Comparison of difference object detection models

Model	Size (MB)	FLOPs (G)	Parameters (MB)	FPS	P (%)	R (%)	F1 (%)	mAP@0.5 (%)
Faster R-CNN	330.3	206.66	41.12	55.6	84.5	84.7	85.0	82.0
SSD	181.1	342.75	23.75	71.4	71.3	83.7	77.0	71.3
YOLOv3	123.4	154.5	61.50	69.9	70.5	68.3	69.4	72.4
YOLOv4	18.7	20.6	9.11	75.2	81.4	81.9	81.7	84.7
YOLOX	16.3	21.6	8.04	68.0	84.3	78.8	81.5	84.6
YOLOv7	74.8	103.2	36.48	62.1	80.1	80.2	80.2	79.2
YOLOv5s	14.4	15.8	7.01	90.9	83.4	80.6	82.0	86.2
PWD-YOLO (ours)	2.7	3.5	1.09	98.0	83.0	82.8	83.0	87.7

In summary, mainstream models are surpassed by the proposed PWD-YOLO in terms of model size, complexity, and detection speed. In terms of detection performance, PWD-YOLO has the best mAP@0.5 compared with other mainstream models, and its F1-score is only slightly lower than that of Faster R-CNN. This indicates that PWD-YOLO can achieve high detection accuracy while maintaining a lightweight model, making it friendlier for deployment in consumer-grade UAVs for real time and rapidly detecting the discolored trees infected with PWD in forest areas, compared to other mainstream models.

**Fig. 9.**
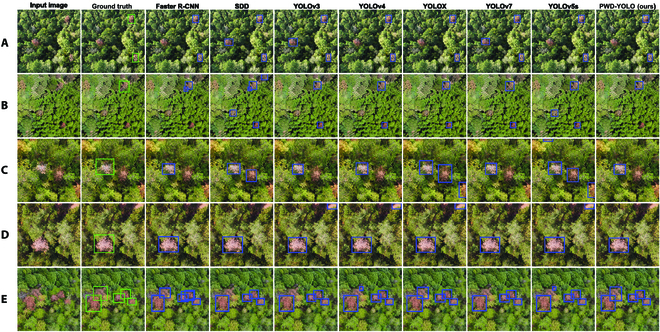
Results of PWD detection by different models in diverse complex environments. (A) Red broad-leaved trees. (B) Red broad-leaved trees. (C) Red broad-leaved trees. (D) Bare land. (E) Intensive target.

To demonstrate the detection performance of each mainstream model more intuitively, the results of each model’s detection were visualized, as shown in Fig. 9. From Fig. 9, it can be seen that discolored trees infected by PWD in the forest can be detected well by PWD-YOLO under any conditions of complex background interference compared with other models, indicating that the model has good robustness.

### Ablation experiments

To provide a clearer demonstration of the improvement process of PWD-YOLO proposed in this study, ablation experiments were conducted, and the results are presented in Table 4. Table 4 shows the performance comparison between ablation studies, including RepVGG, C2fCA module, GSConv, and BiFPN. The improvement process is as follows:

• In the modified M1 model, the original YOLOv5s backbone is replaced with a lightweight RepVGG. When compared to the original YOLOv5s, the detection result on a self-built dataset shows that mAP@0.5 is only increased by 0.2% after the backbone replacement, while the frames per second sees a 28.1 increase from 90.9 to 119.0. In addition, a reduction in both the model size and the number of parameters is observed;
• In this study, a new C2fCA module was designed to be applied in the model’s feature fusion network, enabling the network to have richer gradient flow information while focusing more on the effective features proposed by the network. As shown in Table 4, The C2fCA module is introduced in the M2 model. Compared to the original YOLOv5s, the accuracy is significantly improved by 2.3% to 88.5%, but the model becomes bulkier, and the detection speed is also slowed down;
• The GSConv module was introduced to replace the original C3 module in the feature fusion network, denoted as M3. As demonstrated in Table 4, in comparison to the original YOLOv5s, in M3, an increase of only 0.6% in mAP@0.5 is experienced. However, the model size, computational complexity, and the number of parameters are all reduced to varying degrees, resulting in a simpler model;
• In M4, RepVGG, C2fCA, and GSConv were simultaneously applied. As indicated in Table 4, compared to the original YOLOv5s, notable progress was made in model lightweighting by M4, with an increase of 1.1% in mAP@0.5 reaching 87.3%, but there was no significant improvement in frames per second;
• The BiFPN is applied based on the M4, denoted as M5, further enhancing the network’s feature fusion ability at different scales and improving model detection accuracy. In addition, skip connections were added to the network, and useless feature layers were removed, making the model more lightweight. As shown in Table 4, a noticeable improvement in mAP@0.5 was achieved, reaching 87.7%, an increase of 1.5% compared to the original YOLOv5s, after applying BiFPN. At the same time, an increase of 7.1 in frames per second was also observed, reaching 98.0. Furthermore, the model size, computational complexity, and the number of parameters all decreased dramatically, achieving the goal of model lightweighting, by 5.3 times, 4.5 times, and 6.4 times, respectively, when compared to the original YOLOv5.

**Table 4.** Result of ablation experiment

Model	Improvement strategy				Model size (MB)	FLOPs (G)	Parameters (MB)	FPS	P (%)	R (%)	mAP@0.5 (%)
	RepVGG	C2fCA	GSConv	BiFPN							
YOLOv5s					14.4	15.8	7.01	90.9	83.4	80.6	86.2
M1	√				11.4	15.8	5.52	119.0	82.7	82.0	86.4
M2		√			15.0	16.4	7.30	53.5	86.1	80.9	88.5
M3			√		13.5	15.2	6.57	84.0	81.9	82.5	86.8
M4	√	√	√		2.6	3.4	1.09	91.7	82.8	82.8	87.3
M5 (ours)	√	√	√	√	2.7	3.5	1.09	98.0	83.0	82.8	87.7

**Fig. 10.**
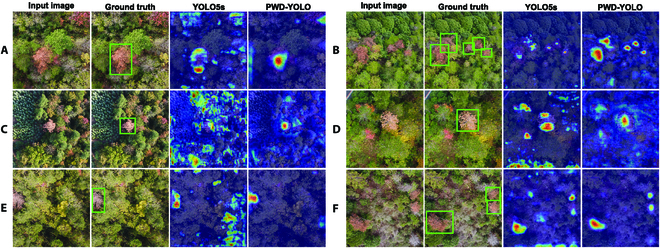
Visualization results of heatmaps for YOLOv5s and PWD-YOLO under different interferences. (A) Red broad-leaved trees and dead trees. (B) Intensive target. (C) Red broad-leaved trees. (D) Red broad-leaved trees and bare land. (E) Incomplete target. (F) Dead trees and bare land.

The feature maps of the model’s network output under different noise interference were visualized using the Grad-CAM tool to demonstrate the detection performance of the proposed PWD-YOLO more clearly, as shown in Fig. 10. According to Fig. 10, it can be clearly observed that the proposed PWD-YOLO in this study can more accurately locate the target and attenuate the interference of background noise compared to the original YOLOv5s. In summary, it is demonstrated by the experimental results that not only was the model made lightweight by our improvement strategy but also the model’s detection performance was enhanced.

### Model evaluation

To further evaluate the generalization capability of the proposed model in unfamiliar scenarios, we conducted evaluations on the test set, and the results are shown in Table 5. In this evaluation, the total number of ground-truth PWD-infected trees in the test set was 2,242. The proposed model detected 2,311 trees, including 2,137 true positives, 174 false positives, and 105 false negatives. The Precision (P), Recall (R), and F1-score (F1) were 92.5%, 95.3%, and 93.9%, respectively. These results indicate that the model demonstrates excellent generalization ability and robustness in different forest environments, making it suitable for detecting discolored PWD-infected trees in various regions.

**Table 5.** Evaluation result of the model on test set

Model	Ground truth	Detected	TP	FP	FN	P (%)	R (%)	F1 (%)
PWD-YOLO	2242	2311	2137	174	105	92.5	95.3	93.9

## Discussion

Accurate and rapid detection of discolored pine trees infected with PWD in complex forest areas is of utmost importance for the prevention and control of PWD [13,14]. Previous studies have demonstrated the feasibility of detecting PWD-infected trees in forest areas by utilizing deep learning methods in conjunction with RGB images captured by UAVs [38,51]. Most forests in southern China consist of mixed forests, rendering them more complex compared to pure forests or plantations. The interconnections among individuals and their relationship with the environment introduce a range of challenges that affect the detection of infected trees. These challenges include vegetation withering, exposed ground surfaces, and the presence of red broad-leaved trees, all of which influence the accurate identification of infected trees [37]. Previous studies have demonstrated that improvements in algorithms can alleviate the impact of complex backgrounds on detection tasks [26,52,53], but these methods typically involve increasing the model size and computational complexity, resulting in slower detection speeds. Furthermore, the resulting large model is not conducive to the deployment of consumer-grade UAVs and similar devices. Therefore, to address the practical needs in forestry management, this study improves the YOLOv5s model based on the recognizable features of PWD-infected trees obtained from close-range UAV RGB images. In this method, the operation of the feature extraction network relies on the lightweight backbone. Unlike Wu et al. [54], our design involves the concatenation of simpler structure RepVGG networks of a single type, avoiding the increased computational costs caused by the flow between different network types. Leveraging its unique residual structure design, it effectively captures crucial texture features of PWD-infected trees. As a result, the feature extraction and inference processes can be carried out more efficiently and rapidly. The issue of channel information loss during computation, caused by lower computation and parameter requirements, is handled within the feature fusion network. The collaboration between the lightweight GSConv network and the C2fCA module, which provides abundant gradient flow information, effectively compensates for the trouble of channel information loss during computation caused by lower computational and parameter requirements. In addition, the BiFPN strategy was adopted to enhance the propagation and sharing of information among PWD-infected trees at multiple scales in the network, which facilitates the rapid extraction of feature information in complex backgrounds, all with scant computational costs. We achieved the frames per second of 98.0 (0.010 s per sheet) with a model size of 2.7 MB in terms of lightweight design. Compared to the methods proposed by Qin et al. [52] (approximately 47 MB and 0.064 s per sheet) and Zhang et al. [53] (approximately 15 MB and 0.010 s per sheet), this method exhibits remarkable advantages. Regarding comprehensive detection performance, the model achieves Precision, Recall, and F1-score values of 92.5%, 95.3%, and 93.9%, respectively, on the test set. Overall, these results surpass the works of Deng et al. (mAP of 89.1%) [40], Hu et al. (Precision of 78.6%, Recall of 95.7%, and F1-score of 86.3%) [41], Xia et al. (F1-score of 83.2%) [55], and Qin et al. (overall accuracy of 79%, Recall of 91%, and Precision of 86%) [42]. Thus, PWD-YOLO possesses distinct advantages in terms of deployment on consumer-grade UAVs for real-time detection during flight in complex forest environments and delivering immediate feedback on detection results, which highlights the significance of the real-time detection method that we have explored.

It should be noted that the advantages of convenience and real-time acquisition offered by UAV remote sensing based on RGB imagery were demonstrated in this study over other close-range remote sensing techniques, which is highly compatible with lightweight object detection models, making it more suitable for close-range monitoring requirements, and can be used as a reliable data source for on-site calibration of satellite and aerial imagery [20]. It is worth discussing the limitations of our proposed method. First, the existing detection method we proposed relies on true-color images captured by RGB cameras, prioritizing the color, texture, and shape features of infected trees. This method is effective in detecting discolored trees with evident symptoms of PWD during the advanced stages of infection [26,56]. Nevertheless, there are certain challenges in accurately distinguishing pine trees infected with PWD using true-color images captured by RGB cameras. On the one hand, various stress conditions like drought, nutrient deficiency, and other diseases and pests can lead to withering and discoloration in pine trees, making it challenging to differentiate them solely on the basis of true-color images. On the other hand, in the early stages of PWD infection, external symptoms are visually indistinguishable, resulting in little disparity between RGB images of infected and healthy pine trees, further complicating the detection process. Therefore, existing research has explored spectral methods, including close-range multispectral and hyperspectral imaging, to address these challenges [57,58]. However, these methods have limitations such as high instrument costs, cumbersome data processing, slow speeds, and lack of real-time capability. Second, the UAV RGB image dataset used in this study only includes samples of masson pine (*P. massoniana*), torch pine (*P. taeda*), and a small number of black pine (*P. thunbergia*) infected with PWD. The model training was based on the features of these samples, which inherently led to limited generalization ability. Furthermore, the dataset used in this study was obtained from UAVs at different flying heights, resulting in distinct variations in spatial resolution. The relationship between spatial resolution and detection accuracy was not specifically considered in the evaluation of the model in this paper.

Therefore, to better meet the practical requirements of forestry management, our future research will primarily concentrate on the following aspects: (a) the rapid detection of PWD-infected trees in the early stages through spectral means, which poses a central challenge; (b) determining the optimal spatial resolution of the image data used for detecting infected trees to maximize the detection performance of this method; (c) incorporating a broader range of infected pine tree varieties into the dataset to enhance the generalization ability of the PWD-YOLO model for other susceptible pine tree species.

## Conclusions

In this study, a real-time and efficient detection method is proposed on the basis of practical demand by improving YOLOv5, and the effectiveness and feasibility of using solely RGB images obtained from UAVs to detect discolored trees infected with PWD in complex mixed forests have been validated. It was found through experiments that the proposed PWD-YOLO maintains the best detection performance of the model while simplifying the network to the greatest extent. The F1-score and mAP@0.5 reached 83% and 87.7%, respectively, which are 1% and 1.5% higher than the original YOLOv5s (82% and 86.2%), demonstrating strong robustness. Compared with other mainstream object detection models, PWD-YOLO has remarkable advantages in terms of model lightweighting, with model size, computational complexity, and parameter size being only 2.7 MB, 3.5 GFLOPs, and 1.09 MB, respectively. The frames per second of 98.0 have been achieved in terms of detection speed, which is friendlier for deployment on embedded devices such as consumer-grade UAVs. When evaluated on the test set, the proposed model achieved Precision, Recall, and F1-score of 93.9%, 92.5%, and 95.3%, respectively. These metrics indicate its excellent generalization capability even across different forest environments. Therefore, as an effective tool, PWD-YOLO is capable of real-time and accurate detection of PWD-infected trees in various settings, providing strong technical support for controlling the spread of PWD and meeting the practical requirements of forest management. This method also offers valuable insights for the application of consumer-grade drones combined with deep learning algorithms in forest resource monitoring, precision forestry, smart forestry, and related areas, demonstrating outstanding prospects for practical applications.

## Data Availability

Some of the data, code and optimal model that were used and analyzed in this study have been uploaded to the website https://github.com/YeXinQuan/PWD-YOLO. In addition, all the homemade datasets in this study (6,308 sheets in total) can be obtained by contacting the corresponding author.

## References

[1] Robinet C, Roques A, Pan H, Fang G, Ye J, Zhang Y, Sun J. Role of human-mediated dispersal in the spread of the pinewood nematode in China. PLoS One. 2009;4(2): Article e4646. 19247498 10.1371/journal.pone.0004646 PMC2645708

[2] Kim N, Jeon HW, Mannaa M, Jeong SI, Kim J, Kim J, Lee C, Park A, Kim JC, Seo YS. Induction of resistance against pine wilt disease caused by *Bursaphelenchus xylophilus* using selected pine endophytic bacteria. Plant Pathol. 2019;68:434–444.

[3] Ikegami M, Jenkins TA. Estimate global risks of a forest disease under current and future climates using species distribution model and simple thermal model–pine wilt disease as a model case. For Ecol Manag. 2018;409:343–352.

[4] Gao R, Wang Z, Wang H, Hao Y, Shi J. Relationship between pine wilt disease outbreaks and climatic variables in the three gorges reservoir region. Forests. 2019;10:816.

[5] Ye JR. Epidemic status of pine wilt disease in China and its prevention and control techniques and counter measures. Sci Silvae Sin. 2019;55:1–10.

[6] Wu W, Zhang Z, Zheng L, Han C, Wang X, Xu J, Wang X. Research progress on the early monitoring of pine wilt disease using hyperspectral techniques. Sensors. 2020;20(13):3729. 32635285 10.3390/s20133729 PMC7374340

[7] Hao Z, Huang J, Li X, Sun H, Fang G. A multi-point aggregation trend of the outbreak of pine wilt disease in China over the past 20 years. For Ecol Manag. 2022;505: Article 119890.

[8] Wang J, Deng J, Yan W, Zheng Y. Habitat suitability of pine wilt disease in Northeast China under climate change scenario. Forests. 2023;14(8):1687.

[9] National Forestry and Grassland Administration. Announcement of pine wood nematode epidemic area. 2023. [accessed 13 April 2023] http://www.forestry.gov.cn/c/www/gkzfwj/380005.jhtml.

[10] Cha D, Kim D, Choi W, Park S, Han H. Point-of-care diagnostic (POCD) method for detecting *Bursaphelenchus xylophilus* in pinewood using recombinase polymerase amplification (RPA) with the portable optical isothermal device (POID). PLoS One. 2020;15(1): Article e0227476. 31935232 10.1371/journal.pone.0227476 PMC6959569

[11] Hao Z, Huang J, Zhou Y, Fang G. Spatiotemporal pattern of pine wilt disease in the Yangtze river basin. Forests. 2021;12(6):731.

[12] Ye JR, Wu XQ. Research progress of pine wood nematode disease. For Dis Insects China. 2022;41:1–10.

[13] Kwon T-S, Shin JH, Lim J-H, Kim Y-K, Lee EJ. Management of pine wilt disease in Korea through preventative silvicultural control. For Ecol Manag. 2011;261(3):562–569.

[14] Syifa M, Park S-J, Lee C-W. Detection of the pine wilt disease tree candidates for drone remote sensing using artificial intelligence techniques. Engineering. 2020;6(8):919–926.

[15] Dash JP, Watt MS, Pearse GD, Heaphy M, Dungey HS. Assessing very high resolution UAV imagery for monitoring forest health during a simulated disease outbreak. ISPRS J Photogramm Remote Sens. 2017;131:1–14.

[16] Stone C, Mohammed C. Application of remote sensing technologies for assessing planted forests damaged by insect pests and fungal pathogens: A review. Curr For Rep. 2017;3:75–92.

[17] Dennison PE, Brunelle AR, Carter VA. Assessing canopy mortality during a mountain pine beetle outbreak using GeoEye-1 high spatial resolution satellite data. Remote Sens Environ. 2010;114(11):2431–2435.

[18] Zhang B, Ye H, Lu W, Huang W, Wu B, Hao Z, Sun H. A spatiotemporal change detection method for monitoring pine wilt disease in a complex landscape using high-resolution remote sensing imagery. Remote Sens. 2021;13(11):2083.

[19] Hicke JA, Logan J. Mapping whitebark pine mortality caused by a mountain pine beetle outbreak with high spatial resolution satellite imagery. Int J Remote Sens. 2009;30(17):4427–4441.

[20] Liang X, Kukko A, Balenovic I, Saarinen N, Junttila S, Kankare V, Holopainen M, Mokroš M, Surový P, Kaartinen H, et al. Close-range remote sensing of forests: The state of the art, challenges, and opportunities for systems and data acquisitions. IEEE Geosci Remote Sens Mag. 2022;10(3):32–71.

[21] Zhang Y, Dian Y, Zhou J, Peng S, Hu Y, Hu L, Han Z, Fang X, Cui H. Characterizing spatial patterns of pine wood nematode outbreaks in subtropical zone in China. Remote Sens. 2021;13(22):4682.

[22] Guimarães N, Pádua L, Marques P, Silva N, Peres E, Sousa JJ. Forestry remote sensing from unmanned aerial vehicles: A review focusing on the data, processing and potentialities. Remote Sens. 2020;12(6):1046.

[23] Iordache M-D, Mantas V, Baltazar E, Pauly K, Lewyckyj N. A machine learning approach to detecting pine wilt disease using airborne spectral imagery. Remote Sens. 2020;12(14):2280.

[24] Yu R, Luo Y, Zhou Q, Zhang X, Wu D, Ren L. A machine learning algorithm to detect pine wilt disease using UAV-based hyperspectral imagery and LiDAR data at the tree level. Int J Appl Earth Obs Geoinf. 2021;101: Article 102363.

[25] Pan J, Lin J, Xie T. Exploring the potential of UAV-based hyperspectral imagery on pine wilt disease detection: Influence of spatio-temporal scales. Remote Sens. 2023;15(9):2281.

[26] Hu G, Wang T, Wan M, Bao W, Zeng W. UAV remote sensing monitoring of pine forest diseases based on improved mask R-CNN. Int J Remote Sens. 2022;43(4):1274–1305.

[27] Girshick R. Fast R-CNN. Paper presented at: Proceedings of the IEEE International Conference on Computer Vision (ICCV); 2015 Jul 07–13; Santiago, Chile.

[28] Ren S, He K, Girshick R, Sun J. Faster R-CNN: Towards real-time object detection with region proposal networks. IEEE Trans Pattern Anal Mach Intell. 2017;39(6):1137–1149. 27295650 10.1109/TPAMI.2016.2577031

[29] He K, Gkioxari G, Dollár P, Girshick R. Mask R-CNN. Paper presented at: Proceedings of the IEEE International Conference on Computer Vision (ICCV); 2017 Oct 22–29; Venice, Italy.

[30] Redmon J, Farhadi A. Yolov3: An incremental improvement. arXiv. 2018. 10.48550/arXiv.1804.02767l.

[31] Bochkovskiy A, Wang C-Y, Liao H-YM. Yolov4: Optimal speed and accuracy of object detection. arXiv. 2020. 10.48550/arXiv.2004.10934.

[32] Wang C-Y, Bochkovskiy A, Liao H-YM. YOLOv7: Trainable bag-of-freebies sets new state-of-the-art for real-time object detectors. arXiv. 2020. 10.48550/arXiv.2207.02696.

[33] Zhu Z, Li D, Hu Y, Li J, Liu D, Li J. Indoor scene segmentation algorithm based on full convolutional neural network. Neural Comput Applic. 2021;33:8261–8273.

[34] Hu W, Huang Y, Wei L, Zhang F, Li H. Deep convolutional neural networks for hyperspectral image classification. J Sens. 2015;2015:258619.

[35] Yu R, Luo Y, Zhou Q, Zhang X, Wu D, Ren L. Early detection of pine wilt disease using deep learning algorithms and UAV-based multispectral imagery. For Ecol Manag. 2021;497: Article 119493.

[36] Pan J, Xie T, You C, Xia X. Dynamic analysis of early stage pine wilt disease in *Pinus massoniana* using ground-level hyperspectral imaging. For Sci. 2023;69(5):529–537.

[37] Sun Z, Wang Y, Pan L, Xie Y, Zhang B, Liang R, Sun Y. Pine wilt disease detection in high-resolution UAV images using object-oriented classification. J For Res. 2021;33:1377–1389.

[38] You J, Zhang R, Lee J. A deep learning-based generalized system for detecting pine wilt disease using RGB-based UAV images. Remote Sens. 2021;14(1):150.

[39] Han Z, Hu W, Peng S, Lin H, Zhang J, Zhou J, Wang P, Dian Y. Detection of standing dead trees after pine wilt disease outbreak with airborne remote sensing imagery by multi-scale spatial attention deep learning and Gaussian kernel approach. Remote Sens. 2022;14(13):3075.

[40] Deng X, Tong Z, Lan Y, Huang Z. Detection and location of dead trees with pine wilt disease based on deep learning and UAV remote sensing. AgriEngineering. 2020;2(2):294–307.

[41] Hu G, Yin C, Wan M, Zhang Y, Fang Y. Recognition of diseased Pinus trees in UAV images using deep learning and AdaBoost classifier. Biosyst Eng. 2020;194:138–151.

[42] Qin J, Wang B, Wu Y, Lu Q, Zhu H. Identifying pine wood nematode disease using UAV images and deep learning algorithms. Remote Sens. 2021;13(2):162.

[43] dos Santos CSS, de Vasconcelos MW. Identification of genes differentially expressed in *Pinus pinaster* and *Pinus pinea* after infection with the pine wood nematode. Eur J Plant Pathol. 2012;132:407–418.

[44] Jocher, G. Ultralytics-YOLOv5. GitHub. 2020. [accessed 2 October 2022] https://github.com/ultralytics/yolov5.

[45] Ding X, Zhang X, Ma N, Han J, Ding G, Sun J. Repvgg: Making VGG-style convnets great again. Paper presented at: Proceedings of the IEEE/CVF Conference on Computer Vision and Pattern Recognition (CVPR); 2021 Jun 20–25; Nashville, TN, USA.

[46] Jocher G, Chaurasia A, Qiu J. YOLO by ultralytics. GitHub. 2023. [accessed 25 January 2023] https://github.com/ultralytics/ultralytics.

[47] Hou Q, Zhou D, Feng J. Coordinate attention for efficient mobile network design. Paper presented at: Proceedings of the IEEE/CVF Conference on Computer Vision and Pattern Recognition (CVPR); 2021 Jun 20–25; Nashville, TN, USA.

[48] Zhang X, Zhou X, Lin M, Sun J. Shufflenet: An extremely efficient convolutional neural network for mobile devices. Paper presented at: Proceedings of the IEEE Conference on Computer Vision and Pattern Recognition (CVPR); 2018 Jun 18–23; Salt Lake City, UT.

[49] Lin T-Y, Dollár P, Girshick R, He K, Hariharan B, Belongie S. Feature pyramid networks for object detection. Paper presented at: Proceedings of the IEEE Conference on Computer Vision and Pattern Recognition (CVPR); 2017 Jun 17–22; Honolulu, HI, USA.

[50] Tan M, Pang R, Le QV. Efficientdet: Scalable and efficient object detection. Paper presented at: Proceedings of the IEEE/CVF Conference on Computer Vision and Pattern Recognition (CVPR); 2020 Jun 13–19; Seattle, WA, USA.

[51] Tao H, Li C, Zhao D, Deng S, Hu H, Xu X, Jing W. Deep learning-based dead pine tree detection from unmanned aerial vehicle images. Int J Remote Sens. 2020;41(21):8238–8255.

[52] Qin B, Sun F, Shen W, Dong B, Ma S, Huo X, Lan P. Deep learning-based pine nematode trees’ identification using multispectral and visible UAV imagery. Drones. 2023;7(3):183.

[53] Zhang P, Wang Z, Rao Y, Zheng J, Zhang N, Wang D, Zhu J, Fang Y, Gao X. Identification of pine wilt disease infected wood using UAV RGB imagery and improved YOLOv5 models integrated with attention mechanisms. Forests. 2023;14(3):588.

[54] Wu Q, Zhang B, Xu C, Zhang H, Wang C. Dense oil tank detection and classification via YOLOX-TR network in large-scale SAR images. Remote Sens. 2022;14(14):3246.

[55] Xia L, Zhang R, Chen L, Li L, Yi T, Wen Y, Ding C, Xie C. Evaluation of deep learning segmentation models for detection of pine wilt disease in unmanned aerial vehicle images. Remote Sens. 2021;13(18):3594.

[56] Jiang X, Wu Z, Han S, Yan H, Zhou B, Li J. A multi-scale approach to detecting standing dead trees in UAV RGB images based on improved faster R-CNN. PLoS One. 2023;18(2): Article e0281084. 36827399 10.1371/journal.pone.0281084 PMC9956600

[57] Yu R, Ren L, Luo Y. Early detection of pine wilt disease in *Pinus tabuliformis* in North China using a field portable spectrometer and UAV-based hyperspectral imagery. For Ecosyst. 2021;8:44.

[58] Wu D, Yu L, Yu R, Zhou Q, Li J, Zhang X, Ren L, Luo Y. Detection of the monitoring window for pine wilt disease using multi-temporal UAV-based multispectral imagery and machine learning algorithms. Remote Sens. 2023;15(2):444.

